# Getting ahead of the ageing curve: learning from EU experiences for a healthier demographic transition

**DOI:** 10.3389/fpubh.2025.1678040

**Published:** 2025-10-17

**Authors:** Hércules Rezende Freitas, João Oliveira Malva

**Affiliations:** ^1^Department of Integrated Medical Sciences, School of Medicine, Rio de Janeiro State University, Rio de Janeiro, Brazil; ^2^Coimbra Institute for Clinical and Biomedical Research (iCBR) CIBB - Center for Innovative Biomedicine and Biotechnology and Institute of Pharmacology and Experimental Therapeutics, Faculty of Medicine, University of Coimbra, Coimbra, Portugal

**Keywords:** demographic transition, healthspan–lifespan gap, geroscience, healthy longevity, policy integration

## Abstract

Population ageing is accelerating worldwide, with one in six people projected to be over 60 by 2030. This demographic shift is already evident in Europe, with slower life-expectancy gains, widening healthspan–lifespan gaps, and growing labour shortages. In this paper, we examine global projections, country-level disparities, evidence from economics, geroscience and public health to assess the conditions under which longevity can generate a net social dividend. Our analysis highlights recent advances in ageing research, including stem-cell, epigenetic and anti-inflammatory interventions, as well as meta-research efforts to promote community-driven consensus on strategic geroscience problems. Translating these insights into societal benefits require regulatory recognition of ageing as a treatable condition, equitable access to innovation, and living-lab ecosystems that connect bench discoveries to health and social care. Using fiscal modelling, we show that disability-driven expenditure could rise by as much as 2.7% of EU GDP annually by 2070, unless offset by slower biological ageing or managed migration. Finally, we suggest that European experiences, particularly from the Iberian Peninsula, can inform South American countries now entering rapid demographic transition.

## 1 Introduction

The 21st century is witnessing the fastest demographic transition in human history. By 2030 one person in six will be 60 or older, and by 2050 this cohort will double to 2.1 billion, while the number of people 80 and over will triple to 426 million (1). This shift is receiving a large contribution from low- and middle-income countries (LMIC), where birth rates have been dropping steeply in the last 45 years. By 2050, 77% of countries (or 151 of 196) are projected to cross below the replacement level (2.1 children per woman) (2). Also, these LMIC are expected to have better public policies foresight to prevent mortality by major chronic diseases (3).

The way high-income regions already ahead on the demographic curve are coping with this challenge offers important insights for action. In the European Union (EU), life expectancy gains, that once felt inexorable, have now started to slow down. England shows the steepest decline, according to an analysis from the Global Burden of Disease (GBD 2021) group (4). Globally, the coronavirus disease 2019 (COVID-19) pandemic underscored the fragility of lifespan; World Health Organization estimates reveal that the crisis wiped out almost a decade of improvement, returning both life expectancy and healthy life expectancy to 2012 levels by 2021 (5). This highlighted the view of international initiatives, such as the United Nations (UN) Decade of Healthy Ageing, which framed longevity as a social dividend that can only be realized through ensured communication, reporting, monitoring and accountability (6).

Recent evidence brings into the spotlight the importance of key strategies to optimize the longevity dividend. A 30-year cohort from the Nurses’ Health Study (1986–2016) and the Health Professionals Follow-Up Study (1986–2016) has shown that higher intakes of fruits, vegetables, whole grains, unsaturated fats, nuts, legumes and low-fat dairy products were associated with a 1.45 to 2.24 odds ratio (OR) for healthy ageing, depending on dietary pattern and stratification (7). In addition, an 18-year cohort of the Australian Longitudinal Study of Women’s Health detected an OR of up to 1.59 for falls amongst women with physical activity levels smaller than 150 min a week (8).

Promoting diet and physical activity alone, however, is insufficient to deal with the complexity of ageing on a populational scale. Advancing the healthspan also requires reframing global ageing as an opportunity to create new systems of care, applying implementation science, addressing comorbidities, evaluating costs and benefits, using advanced analytic methods with up-to-date data, and including underrepresented populations (9, 10).

**Figure 1:**
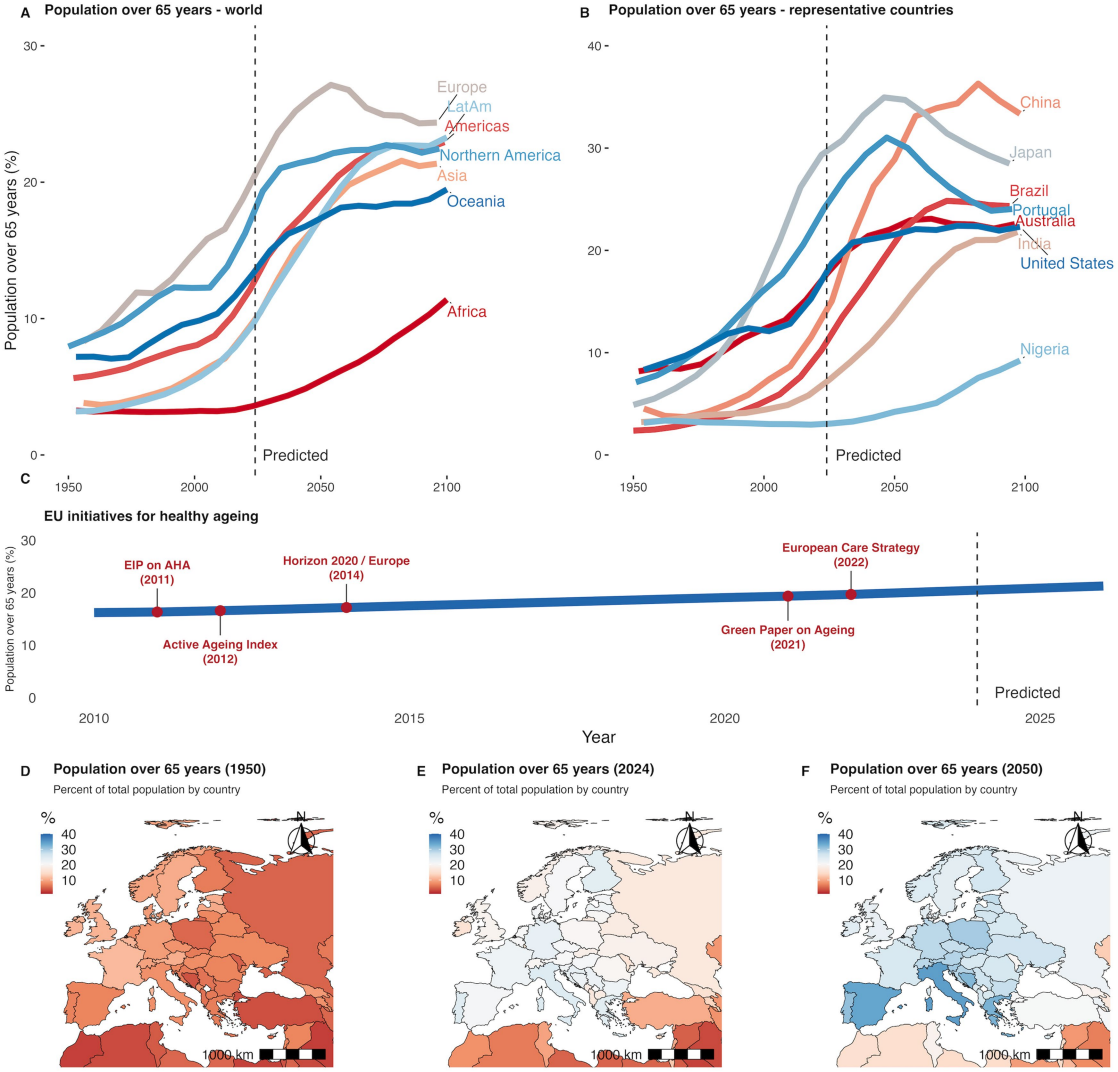
Population ageing worldwide and EU initiatives. **(A)** Population 65 and over as a percentage of the total population by world region, from 1950 to 2100. **(B)** Population 65 and over in selected countries representing regional diversity. Solid lines indicate historical data; dashed vertical line marks the beginning of projections. **(C)** Timeline of key European Union initiatives to promote healthy ageing, indicating the year and name of each policy or program. **(D)** Population 65 and over as a percentage of the total population by country in 1950. **(E)** Percentage of population 65 and over in 2024. **(F)** Projected percentage in 2050. Data are shown by country and represented using a diverging colour scale from low (red) to high (blue). Data from UN, World Population Prospects (2024) (40).

In this work, we argue that longevity can yield a net social dividend when basic science and public policy are developed in concert and continually calibrated to one another. Figure 1 brings the whole trajectory into view, moving from worldwide numbers to country-by-country detail and, finally, to the European policy milestones that respond to those trends.

An older population will have a dramatic impact on how the society is organized. Recent models show that the migration towards an older labour force can impact overall economic development and gross domestic product (GDP) per capita growth (11). Physical labour poses an additional concern. Population-data analysis shows that an older farming workforce is associated with decreased agricultural production (12). These results highlight the importance of supporting a healthier transition to late working life. A recent systematic review has developed a seven-step framework to better incorporate the older workforce, especially into highly technological environments (13). Similar systems can be developed to prepare other sectors for this new demographic. The same demographic headwinds strain clinical capacity.

Low-quality ageing also weighs heavily on health systems. Over 60% of individuals over 85 years in the United States (US) live with two or more chronic conditions. These individuals also account for most healthcare expenditures, often requiring complex, continuous, and multidisciplinary care. These challenges can be worsened by systemic inequalities, magnified in rural, low-income, and underserved communities (14). Public health research highlights the importance of restructuring of the healthcare system to favour integrated care models, with home and community-based services, equity-centred policy reforms, and investments in geroscience and digital health (15).

Demographic compensation is also posed as a strategy to mitigate the loss of younger workforce. This idea, however, is faced with many challenges, as uncontrolled migration may impose equally complex social adaptations. A recent analysis of Organisation for Economic Co-operation and Development (OECD) data has shown that immigrants tend to substitute younger native workers, and that adult natives usually benefit from this complimentary increase in workforce. Also, the European Commission Ageing Report of 2024 has estimated that a higher migration scenario will decrease the age-related GDP spending by, on average, −0.52% per year until 2070. These findings highlight the need for age-specific policies and programs to allow for an effective immigration-centred demographic compensation strategy (16, 17).

Some non-EU countries, however, also face a critical scenario. Japan already has a proportionally older population that is greater than the highest EU prediction (for 2056), and China will reach an even higher rate by 2085. While other regions are expected to have a smaller older population, most countries will have a fifth of the population older than 65 years by 2068. These numbers highlight the importance of initiatives focused on healthy ageing, both in terms of scientific efforts and public policies.

As the most impacted region, the EU has promoted several strategies to deal with this demographic shift. Beginning in 2011 with the European Innovation Partnership on Active and Healthy Ageing (EIP on AHA), the EU has layered successive frameworks, including: (a) the 2012 Active Ageing Index that quantified progress; (b) the 2021 Green Paper that reframed ageing as a life-course challenge; (c) the 2022 European Care Strategy that translated that vision into service standards. This roadmap was supported by financial instruments for demonstrator projects, first through Horizon 2020 and now through Horizon Europe, so that metrics, policies and innovations evolve in lockstep towards healthy longevity.

This study builds on these European experiences to address two core motivations: the need to understand how longevity can deliver a net social dividend and the urgency of supporting regions, such as South America, that are now entering rapid demographic transition. First, we synthesize global and regional demographic trends, then review scientific advances and meta-research in geroscience, assess fiscal and policy challenges through economic modelling, and finally propose a set of strategic recommendations inspired by European roadmaps that can inform South American policy and research agendas.

## 2 Healthspan and translational research

These demographic forecasts underscore why closing the healthspan gap is now imperative. Population-level data now leave little doubt that simply adding years is no longer enough. Between 2000 and 2019 global life expectancy rose by 6.4 years, yet healthy life expectancy, measured as years lived free from significant disability, grew by just 5.3 years. That strain is visible in the healthspan–lifespan gap: extra years are still disproportionately burdened by morbidity (5). In Europe the picture is equally dichotomous: the OECD’s 2024 “Health at a Glance” review highlights wide national divergences, with some Member States gaining barely 2 months of healthy life per calendar year while others lose ground altogether (18). Closing this healthspan–lifespan gap demands upstream prevention, strong translational research, equitable access to innovation and systematic tracking of functional capacity over the life course.

In terms of translational research, mechanistic work on healthy longevity converges on two inter-locking pathways. The first curbs damage accumulation, recalibrating energy sensing through caloric-restriction mimetics, AMPK/SIRT activation or mTOR inhibition and sweeping mis-folded proteins via autophagy, thereby protecting metabolism, proteostasis and macromolecular integrity; the second restores systemic resilience by enhancing regeneration and tempering stress-inflammatory circuitry, as stem-cell replenishment, epigenetic reprogramming (19), mitohormetic conditioning and senomorphic anti-inflammatories quell chronic insults and sustain tissue function (20).

While these research avenues show promising results, new strategies can be implemented to optimize the identification of literature gaps and poorly explored ideas. A recent preprint by Magalhães proposes an updated view of the classic Strehler’s list of open questions on cellular ageing (21). Their approach combined community-driven input, workshop collaboration, and natural language processing to pair over 204 open problems in biogerontology, contributed by the community, against 200,228 articles found on PubMed under the MeSH term of “Ageing.” Then, a final list of 100 problems was selected and organized into 11 categories, providing a data-driven consensus of broad philosophical questions and concrete, testable hypotheses, that can be used to shape the future of ageing research (22).

Yet even as mechanistic insight deepens, regulatory frameworks still assume ageing is not an addressable condition. While some strategies are easily implemented, especially those related to lifestyle and preventive health (23), the widespread adoption of anti-ageing drugs or supplements in healthcare is more challenging. Most regulatory bodies, including the European Medicines Agency, impose strict standards for new anti-ageing pharmacological therapies, not recognizing ageing as a disease (24, 25). While this ensures a high level of safety, authors have pointed that it may generate several false negatives, which would leave potentially effective resources out of reach for ample medical use (26). In contrast, access to healthcare and anti-ageing treatments is highly dependent on socio-economic status. Countries with low economic heterogeneity may face a smaller obstacle than those with high wealth inequality, such as South Africa, Brazil, and the US. In these countries, access to socialized medical care may be a determining factor for healthy ageing (27).

To overcome precisely such translational bottlenecks, Europe has spent the past decade constructing a translational ecosystem that now ranges from basic biology to market deployment. The EIP on AHA pioneered the model: its Reference Sites bring together hospitals, regional governments, SMEs, civil society and academia to demonstrate at-scale solutions for healthy longevity and to share evidence through the Monitoring and Assessment Framework for the EIP on AHA (MAFEIP) (28). Horizon Europe continues the trajectory; the 2025–27 Health Work Programme explicitly earmarks funds for living-lab trials of advanced therapies, AI-enabled decision support and environmental determinants of brain ageing, all tied to measurable quality-of-life endpoints (29). Joint Programming Initiatives such as “More Years, Better Lives” add a policy lens, coordinating national research agendas on labour-force shortages in long-term care and other pressing demographic challenges (30). Together these platforms shorten the journey from bench to bedside and, crucially, from pilot to procurement.

## 3 Involving citizens and policymakers

Digital transformation has turned older citizens from passive recipients into data producers and co-researchers. The EU-funded “Every Walk You Take” initiative in Barcelona, for example, co-creates a m-health platform that lets volunteers over 55 map personalized walking routes using real-time air-quality and micro-climate data, simultaneously generating city-scale evidence on environmental exposures and physical activity (31). Pan-European infrastructures such as SHARE, the Survey of Health, Ageing and Retirement in Europe, complement these bottom-up efforts with harmonized longitudinal datasets that are openly accessible to academics and policymakers alike (32). Progress towards the Digital Decade target of universal electronic health records by 2030 will further democratize access, provided safeguards on privacy and digital literacy accompany rollout (33).

Science alone, however, cannot realign societies around longevity; coherent policy is indispensable. The European Commission’s 2021 Green Paper on Ageing reframed ageing as a continuum that begins in childhood and intersects with education, housing, employment and climate resilience. By adopting a life-course approach, the Paper invites ministries of health, finance, transport, digital, and labour to co-own the agenda and to design prevention-oriented regulation (34). Reference Sites operationalize that vision by embedding regional policymakers in quadruple-helix teams (i.e., public institutions, academia, industry, and civil society), ensuring that research findings translate into procurement frameworks, building codes and reimbursement schedules. The result is a feedback loop in which policy signals guide research priorities and, conversely, real-world evidence refines policy instruments. These co-creation models foreshadow the systems that will define an age-integrated society after 2050.

Ageing@Coimbra exemplifies a dynamic regional approach to addressing the complex challenges of population ageing through the integration of scientific research, public health strategy, and community engagement. Anchored in the Quadruple Helix model, linking academia, government, industry, and civil society, the initiative nurtures innovation in active and healthy ageing by involving over 90 partners from the Centre Region of Portugal. Supported by key institutions, such as the Multidisciplinary Institute of Ageing (MIA-Portugal), while coordinated with the Commission for the Coordination and Regional Development of the Centre (CCDRC), Ageing@Coimbra has helped design public health strategies that are both evidence-based and responsive to demographic and territorial realities. This collaborative model has led to the development and implementation of tools for the evaluation of healthy lifestyle for the general public (35). In recognition of its impact, the CCDRC established the Good Practices Award in Active and Healthy Ageing, which in 2017 celebrated initiatives that successfully connected health, lifestyle, and knowledge to improve the lives of older citizens and promote sustainable regional development (36).

## 4 A long-term view on ageing

Looking beyond 2050, when one in six people worldwide will be over 65 and global population growth will plateau, societies that thrive will be those that treat longevity as a design parameter for every system, from housing stock to fiscal policy. Climate change, urbanisation and technological disruption will interact with demographic trends, amplifying inequality unless health, social and economic policies converge on the shared objective of extending healthy, productive years.

Addressing the main causes of disabilities is a key action to help sustain a more active life in later age groups. An analysis of the Dunedin cohort has used 19 biomarkers to determine a pace of ageing (PoA) for participants at ages 26, 32, 38, and 45 years. This measure was then associated with ageing outcomes across 4 domains, including brain age, cognitive measurements, sensorimotor functional capacity, and perceptions of ageing. Authors have shown that, at 45 years of age, PoA displays a positively skewed distribution, with most people ageing at a rate of 0.5 to 1.5 years of physiological change per chronological year, and a smaller number of participants ageing at a rate of 1.5 to 2.5 years per year. These results strengthen the idea that improving function will outweigh the concerns over chronology in the future of ageing (37).

**Figure 2:**
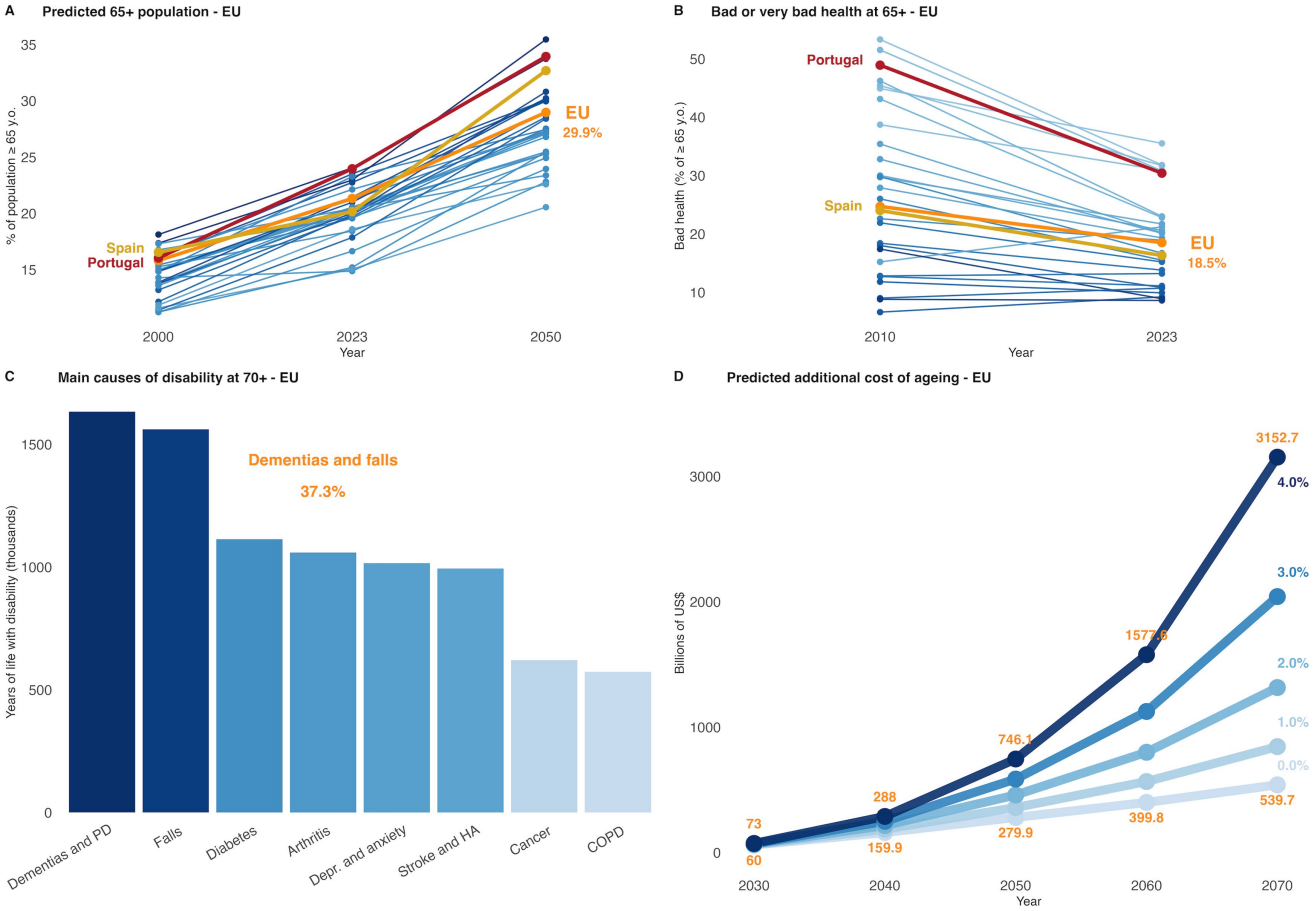
Population ageing, disability factors, and economic impact in the EU. **(A)** Based on data from Eurostat, the OECD suggests that, by 2050, three in 10 people in the EU will be over age 65, up from two in 10 in 2023. Projections show the EU reaching 29.9% of individuals over the age of 65 by 2050. **(B)** From 2010, most EU countries reported a decrease in the percentage of people over 65 years reporting bad or very bad health. Germany (+6%), Ireland (+2.6%), Sweden (+1.7%), and Malta (+0.4%) reported an increase in bad health reports. **(C)** Dementias, Parkinson’s disease (PD), and falls are the main causes of years of life with disability amongst EU residents order than 70 years (in 2021). Combined, the three main factors comprise of almost 40% of the total time spent with disabilities, when considering most impactful causes. Depr., depression; HA, heart attack; COPD, chronic obstructive pulmonary diseases. Data from the OECD “Health at a Glance: Europe 2024” report. **(D)** Predicted additional public expenditure on ageing in the European Union from 2030 to 2070, based on the 2024 Ageing Report (risk scenario). Each line represents an alternative GDP growth trajectory, from 0.0% (lightest blue) to 4.0% (darkest blue). Projections include long-term costs associated with pensions, health, and long-term care systems. Higher GDP growth rates are associated with a proportionally lower fiscal burden of ageing relative to total economic output. Point labels represent estimated total costs in billions of US dollars. Scenario assumptions are based on constant age-related spending profiles and demographic projections provided by Eurostat (17).

What are, then, the major disability components to prevent at an earlier age? As shown in Figure 2, 30% of the EU population is expected to be over 65 in 2025. As of 2023, however, not all countries were able to advance in terms of improving the self-reported perception health in this age group. To address this problem, the development of preventive strategies, and treatment, focused on key disability factors such as dementias, Parkinson’s disease, falls, diabetes, arthritis, psychiatric and cardiovascular diseases, will be fundamental for a sustainable older society. Concerns over the financial burden involving research, education, and public health services to prevent these disabilities were also addressed by the 2024 Ageing Report (European Commission). In the risk scenario, with high demand for healthcare & long-term care, ageing will cost up to 2.7% more than the baseline prediction. Considering GDP growth predictions of zero to 4 %, from $19.99 trillions in 2025, a high rate of disability will cost an additional $0.54 to $3.15 trillions a year by 2070.

## 5 Discussion

Ageing does not happen equally for all individuals, nor does it emerge in the same way for different countries or cultures. Both research and social experience have shown that the path for a healthier ageing, even a longer one, will demand forward-thinking policy, with sustained investment in geroscience, as well as in technologies that are eventually incorporated into public health programmes.

Also, the differential rate of ageing between the major geographical regions will require a new view on migration and international partnerships. Based on the latest projections, the economic sustainability of countries such as Japan, China, Portugal, Spain, Italy, and the US will depend on strategic migration and reduced PoA for the older adult population.

The Active and Healthy Living platform now evolving out of the EIP on AHA provides a practical template: it couples region-to-region knowledge exchange with a commitment to open standards, thereby enabling innovations developed today to remain interoperable with the needs and technologies of tomorrow. It also aims at modernising social protection and fostering legal migration and integration as part of a policy mix.

In that spirit, translating science into policy is not a one-off relay but a permanent co-evolution, requiring vigilance, foresight and an unwavering emphasis on equity across the life course. From this perspective, and with attention to historical and cultural specificities, South American countries facing emerging demographic transition may profit from policies implemented in Europe, particularly in Iberian Peninsula, to reduce the expected burden on health and social care systems.

The transferability of public policies on healthy ageing from Europe to South America depends on a complex interplay of institutional capacity, political legitimacy, economic feasibility, and cultural alignment. As highlighted by Ghența et al. (38), the successful adaptation of practices for older adults requires more than a technical appraisal of what worked in the European context; it demands systematic evaluation of local administrative resources, financial sustainability, and the involvement of local stakeholders who can ensure social acceptance (38). Similarly, Stone et al. (39) emphasizes that policy transfer is never a neutral process of lesson drawing but is shaped by the circulation of ideas, the influence of international norms, and the power dynamics between donor and recipient contexts.

Applied to the domain of healthy ageing, this means that while European models of integrated care, social inclusion, and community-based health services may offer valuable insights, their adoption in South American countries must consider existing health system capacity, political commitment, and societal attitudes towards ageing. Without such adaptation, policy transfer risks being superficial, reinforcing dependency rather than fostering genuine local innovation in ageing policy.

## 6 Conclusions, limitations, policy implications, and future research directions

Our perspective underscores that while the global challenge of ageing calls for shared frameworks and cooperative solutions, policies must remain deeply attuned to the historical, cultural, and institutional realities of each region.

A limitation of this analysis is that it draws primarily on European and South American experiences, which means that the diversity of demographic transitions in Africa and Asia remains to be explored in future work. The policy implications, however, are evident: sustainable investment in geroscience, digital health technologies, and managed migration must be combined with local innovation, political commitment, and stakeholder engagement to ensure resilience in the face of demographic change.

Future research should therefore investigate how different institutional capacities and social attitudes shape the adaptation of healthy ageing policies, while also developing frameworks for equitable knowledge exchange that transform international collaboration into a process of co-evolution rather than unilateral transfer.

The demographic transition demands policies that are both evidence-based and adapted to local realities, with European lessons serving as guidance rather than fixed models. When coupled with innovation and international collaboration, these transfers can help South American nations ease future pressures on health and social care, making clear that the only way to get ahead of the ageing curve is to start now.

## Data Availability

The original contributions presented in the study are included in the article/Supplementary material, further inquiries can be directed to the corresponding author.
